# Left Ventricular Pseudoaneurysm Following Inferior Myocardial Infarction: A Case for Conservative Management

**DOI:** 10.14740/cr449w

**Published:** 2016-02-20

**Authors:** Jonathan Ludmir, Karan Kapoor, Praveen George, Jasjeet Khural, Brian Barr

**Affiliations:** a University of Maryland Medical Center, Baltimore, MD, USA

**Keywords:** Pseudoaneurysm, Left ventricular mass, Echocardiography

## Abstract

Left ventricular pseudoaneurysm is a rare complication of myocardial infarction that carries a high mortality rate. Although conventional wisdom suggests prompt surgical repair in order to mitigate risk of expansion and rupture, there are some data to support non-operative management in asymptomatic individuals with likely chronic pseudoaneurysms, particularly when surgical candidacy is poor. We present a case of a medically managed left ventricular pseudoaneurysm subsequent to inferior ST-segment elevation myocardial infarction with 6-month follow-up data.

## Introduction

Left ventricular pseudoaneurysm is a rare complication of myocardial infarction (MI) associated with serious morbidity and mortality [1]. Unlike true ventricular aneurysms in which the integrity of the myocardial wall is maintained, pseudoaneurysm forms when cardiac rupture is contained by adherent fibrous tissue and pericardium. Although challenging to diagnose, advances in non-invasive imaging have enhanced identification of this condition and increased detection of incidental pseudoaneurysms in otherwise asymptomatic individuals [2]. The natural history of untreated left ventricular pseudoaneurysm is based predominantly on retrospective single-center case series and remains largely undefined. Left ventricular pseudoaneurysms render a 30-45% risk of rupture [1]. However, there are retrospective data that suggest a lower risk of rupture amongst a subset of chronic pseudoaneurysms [3]. Concomitantly, post-operative mortality after surgical repair of left ventricular pseudoaneurysm is high, ranging from 23% to 50% in some series [2, 4, 5]. We present a case of left ventricular pseudoaneurysm following inferior ST-elevation MI.

## Case Report

**Figure 1:**
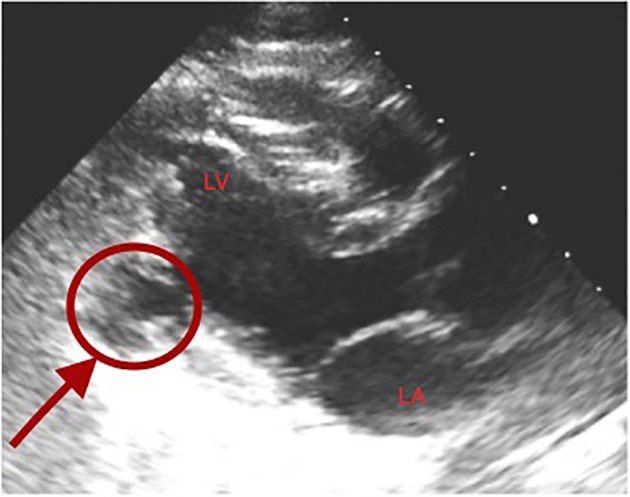
Transthoracic echocardiogram demonstrating left ventricular pseudoaneurysm (arrow) in the distal inferolateral wall.

**Figure 2:**
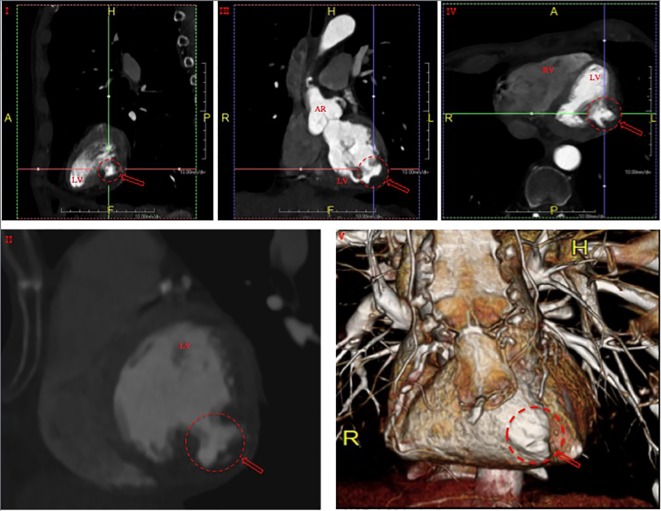
Cardiac computed tomography demonstrating a 1 × 2 cm outpouching along the inferolateral left ventricular wall at the mid cavity level in sagittal (I, II), coronal (III), axial (IV) and three-dimensionally reconstructed (V) views, consistent with pseudoaneurysm (arrows). RV: right ventricle; LV: left ventricle; AR: aortic root.

A 68-year-old man with history of chronic obstructive pulmonary disease and asbestos-related pneumoconiosis presented with chest pain and diaphoresis after a motor vehicle accident. Electrocardiogram revealed ST elevations in the inferior leads. The patient was promptly taken to the cardiac catheterization lab where he was found to have complete occlusion of the distal right coronary artery. The patient underwent successful percutaneous coronary intervention of the right coronary artery with placement of a drug eluding stent. One day after the coronary intervention, he remained asymptomatic when echocardiogram revealed a left ventricular pseudoaneurysm in the distal inferolateral wall (Fig. 1). Left ventricular ejection fraction (LVEF) was 40%, and no valvular abnormalities or pericardial effusion were detected. The patient subsequently underwent cardiac computed tomography which confirmed the diagnosis. A 1 × 2 cm outpouching was visible along the inferolateral left ventricular wall at the mid cavity level, consistent with a pseudoaneurysm (Fig. 2, Supplementary video 1, www.cardiologyres.org). In light of his underlying pulmonary disease conferring a high surgical risk, the patient opted for medical therapy alone with an angiotensin converting enzyme inhibitor. At 6 months follow-up, the patient remains asymptomatic and his echocardiogram is unchanged.

## Discussion

A rare complication of either transmural MI or cardiac surgery, acquired pseudoaneurysm of the left ventricle, poses a difficult management dilemma, particularly among hemodynamically stable patients who are asymptomatic yet poor surgical candidates. The risk of expansion and eventual rupture must be balanced against a high surgical mortality, but the rarity of the condition has made understanding its natural history and identifying the highest risk patients challenging.

Risk factors for development of left ventricular pseudoaneurysm include female sex, first occurrence of either lateral or anterior wall MI, age greater than 60 years and severe single-vessel coronary artery disease, the latter two of which were features in our patient [6]. The clinical presentation is also highly variable, ranging from asymptomatic (up to 50% in one series) to syncope, arrhythmia, heart failure or severe angina [3]. Transthoracic echocardiography is widely regarded as an appropriate initial diagnostic assessment, although this may be followed by cineventriculography or cardiovascular magnetic resonance imaging given the high false negative rate associated with the former [7, 8]. Risk factors for eventual rupture have not been clearly delineated, although larger size, posterolateral location and poor collateral circulation have all been suggested as potential predictors [2]. Notably, time till development of pseudoaneurysm does not reliably predict risk of expansion, as both early (within 48 h) and late (greater than 48 h) post-infarction pseudoaneurysms have similar rates of rupture [6, 9, 10].

Conservative management of left ventricular pseudoaneurysms has been described in several case series (Table 1 [1, 3-5, 11]). Natarajan and colleagues reviewed a total of 66 cases of post-infarction pseudoaneurysm published between 1984 and 1993, demonstrating that medical management of chronic pseudoaneurysm (diagnosed 3 or more months following MI) was not associated with an increased risk of rupture [12]. In the largest series to date, Frances et al reported a 48% (15 of 31 patients) mortality within 1 week of pseudoaneurysm diagnosis [1]. However, the remaining 16 medically managed patients had median survival of 3 years. The authors speculated the high mortality rate to relate to the relatively large size of pseudoaneurysm in their series (median posteroanterior diameter of 6 cm, median orifice diameter of 2 cm). Furthermore, 40% of the conservatively managed cohort was lost to follow-up. Thereafter, a cohort of conservatively managed patients from the Mayo Clinic experienced an overall 2-year survival rate of 63% [3]. Among the six medically treated patients who eventually died beyond this initial 2-year period, three died of non-cardiac causes, two of pre-existing congestive heart failure and one of acute MI. Importantly, no patient experienced further cardiac rupture. Most recently, Moreno et al reported an 1- and 4-year survival rate of 88.9% among nine conservatively managed post-infarct pseudoaneurysm patients, and no progression to rupture in any case [11]. Interestingly, the cumulative incidence of ischemic stroke was high in these individuals (10% at 1 year and 33% at 4 years), although the issue of chronic anticoagulation in medically managed patients remains controversial. Of importance is the fact that compared to other series, the average size of pseudoaneurysm in this study was relatively small (mean posteroanterior diameter of 29.1 mm, mean orifice diameter of 9.8 mm). Furthermore, unlike in our case, median time to pseudoaneurysm diagnosis in these studies ranged from 3 to 9 months [1, 3, 11].

**Table 1:** Select Case Series Reviewing the Natural History and Treatment Outcomes of Left Ventricular Pseudoaneurysms

Authors	Study type	n	Age, years	Type and location, %	Causes of pseudoaneurysm, %	Management type, n	Follow-up period	Mortality findings
Yeo et al, 1998 [3]	Retrospective case series	52	Mean 48 ± 28	Left ventricle Inferior/posterolateral, 35 Sub-MV, 8 Subaortic, 6 Right ventricle RVOT, 25	Surgery, 58 MI, 42	Surgical, 42 Medical, 10	Median of 2.3 years	Surgical mortality 31% at 2.3 years Medical 60% at 2.3 years (no deaths due to rupture)
Frances et al, 1998 [1]	Systematic review	244	Median 60	Left ventricle Posterior, 43 Lateral, 28 Apical, 24 Inferior, 19 Anterior, 18 Basal, 14	MI, 55 Surgery, 33 Trauma, 7 Infection, 5	Surgical, 193 Medical, 51	1 - 10 years	Surgical post-operative mortality < 3 days 23%, remainder alive till about 4 years; Medical mortality at < 1 week, 48%, 61% at 1 year, 84% at 5 years, 94% at 10 years
Pretre et al, 2000 [5]	Retrospective case series	10	Mean 66	Left ventricle Inferior, 80 Lateral, 10 Anterior, 10	MI, 70 Surgical, 30	Surgical, 10	Median 45 months	Surgical mortality 28% in post-MI group; overall 30%
Moreno et al, 2003 [11]	Retrospective case series	10	Mean 63 ± 12	Left ventricle Inferior, 6 Anterior, 3 Anterior/inferior, 1	MI, 10	Surgical, 1 Medical, 9	Mean 3.8 years	Medical mortality at 4 years 10%
Atik et al, 2007 [4]	Retrospective case series	30	Mean 68 ± 8	Left ventricle Posterior, 39 Lateral, 29 Inferior, 21 Anterior, 11	MI, 100	Surgical, 30	Mean 52 ± 37 months	Surgical mortality 20% immediate and 27%, 41%, and 55% at 1, 5, and 8 years

Our patient’s severe underlying pulmonary disease precluded aggressive surgical management. Although possible, it is unlikely that his pseudoaneurysm was chronic and diagnosed incidentally on a routine post-infarct echocardiogram, which was not obtained under a high pre-test suspicion for pseudoaneurysm. As such, his clinical profile - an asymptomatic, acute and moderately sized pseudoaneurysm - reflects a slightly different demographic than captured by the aforementioned series, and makes application of a robust evidence-based management decision challenging. Development of a centralized registry of cases may help to better elucidate the true natural history of this rare entity, and should be the focus of future studies especially as advances in non-invasive diagnostic modalities are made and diagnostic awareness increases.
